# Mechanism-aware and explainable artificial intelligence for drug release prediction in polymeric drug delivery systems

**DOI:** 10.3389/fmed.2026.1975727

**Published:** 2026-09-23

**Authors:** Mohammed Ghazwani, Yahia Alghazwani, Umme Hani

**Affiliations:** 1 Department of Pharmaceutics, College of Pharmacy, King Khalid University, Abha, Saudi Arabia; 2 Department of Pharmacology, College of Pharmacy, King Khalid University, Abha, Saudi Arabia

**Keywords:** drug release modeling, interpretable artificial intelligence, PLGA nanoparticles, polymeric drug delivery systems, temporal representation learning

## Abstract

Predicting drug release from polymer-based delivery systems remains challenging because release behavior depends on nonlinear interactions among formulation composition, polymer properties, drug characteristics, processing conditions, and time. This study develops an interpretable artificial intelligence framework for predicting cumulative drug release from PLGA nanoparticle formulations prepared by nanoprecipitation. A curated dataset containing 4,909 formulation–release observations was used to integrate formulation-informed feature engineering, temporal representation learning, symbolic regression, latent-space analysis, and explainable machine learning. The proposed framework achieved *R*^2^ = 0.9977, RMSE = 1.56 × 10^−4^, and MAE = 1.31 × 10^−4^ on the training data, while the independent test set yielded *R*^2^ = 0.9975, RMSE = 1.64 × 10^−4^, and MAE = 1.31 × 10^−4^. Y-scrambling produced substantially lower *R*^2^ values of 0.0636–0.0714, providing evidence that the observed predictive performance is not readily reproduced after randomization of the response. Temporal autoencoder representations further revealed structured organization of release trajectories in a low-dimensional latent space. Nevertheless, the high predictive accuracy should be interpreted cautiously because the dataset contains repeated observations associated with formulation–release trajectories and includes release time as an input-related variable; therefore, random observation-level splitting may provide more favorable estimates than formulation-level or study-level external validation. The framework provides a computational basis for formulation screening and hypothesis generation, but experimentally validated release experiments and prospective external datasets remain necessary before practical deployment.

## 1 Introduction

Drug release from polymeric delivery systems is a complicated task that requires knowledge of many interacting formulation and material factors, such as polymer properties, particle size, drug loading, encapsulation efficiency, degradation behavior, diffusion mechanisms, and environmental conditions (1). The resulting release profiles often demonstrate nonlinear temporal behavior such as burst release, sustained release, diffusion-controlled transport, and saturation dynamics. Therefore, designing and optimizing drug delivery systems typically involves a lot of experimental work that is expensive and time-consuming (2). Machine learning advances have brought about the capability to predict with high accuracy pharmaceutical properties and release behavior from formulation data. However, having only accurate prediction models is usually not enough; the learned models also need to be interpretable (3). Most of the existing ML methods for drug release prediction are based on the use of complex black-box models whose internal decision-making processes are not logically or mathematically traceable in a succinct manner (4, 5). Such models may have very good predictive performance, but their limited interpretability leaves them incapable of providing mechanistic understanding, scientific insight, and regulatory confidence (6). In pharmaceutical and healthcare applications, transparent models are of great value because researchers and practitioners want to understand how the input variables affect the system behavior rather than just getting accurate predictions (7, 8).

SR has recently emerged as a very promising branch of machine learning whose main objective is to figure out symbolic mathematical expressions straight from data. One of the main hypothesis-generating tools in symbolic regression is to find a symbolic expression that faithfully summarizes the characteristics of a dataset (9). Compared to traditional predictive models, symbolic regression identifies the actual mathematical formulas that show the relationships between the input variables and the target responses (10). As scientific areas get more data-heavy, interpretable models that can support scientific discoveries are in increasing demand. Even though there have been advancements in machine learning and mechanistic modeling of drug release systems, symbolic artificial intelligence for direct discovery of closed-form drug release equations has been explored by only a few studies (11, 12). Besides, these approaches hardly ever combine temporal release learning, mechanistic constraints, latent behavior discovery, and symbolic equation extraction within a single framework. So, a mechanism-aware symbolic AI framework is still needed that can produce physically meaningful, interpretable, and scientifically informative drug release equations (13).

Traditionally, figuring out precisely how a drug tablet behaves over time within the body has involved a fair bit of trial and error in the lab and can be a time-consuming process (14). However, recent innovations in artificial intelligence have drastically enhanced the accuracy of forecasting drug-release patterns from various pharmaceutical systems. Protopapa and colleagues (15) found that with machine learning, one can not only predict the entire drug dissolution profile but also determine the kinetic parameters from the mere knowledge of tablet compositions alone. This path-breaking study reveals that data-driven methods have the potential to minimize experimental trials significantly. Similarly, Sun and co-workers (16) brought together machine learning and lab work to discover which variables influence drug release from PLGA nanoparticles most, and Mahdi et al. (17) took it a step further and found that the high-dimensional spectral and formulation data are highly useful for release prediction. Despite the great promise of purely data-driven methods, a good number of researchers have taken a step back and re-examined mechanistic modeling of release processes as a potential path. D'Ambrosio et al. (18) came up with models that can explain different nanogel release phases, which depend on the environmental conditions they are subjected to. Alshahrani et al. (19) took mechanistic mass transfer concepts and mixed them with machine learning in order to make predictions of polymer matrix drug release kinetics more accurate. The mechanistic studies by Vasileiadou et al. (20), Kalosakas (21), and Jain et al. (22) pinpointed the roles of drug release controlling factors such as degradation, diffusion, bond cleavage, and dissolution-front propagation. Their work has offered the scientific community an understanding of the physics behind the release mechanisms. Interpretability is one of the key aspects of AI that has recently attracted researchers’ attention. Wahlquist et al. (1) showed that symbolic regression networks can be used to automatically find interpretable pharmacometric equations without sacrificing the prediction performance. Similarly, Bannigan et al. (23) used machine learning to facilitate the design of polymeric long-acting injectables through accurately predicting the long-term release behavior. These findings notwithstanding, the bulk of the work to date has been focused on optimizing predictive accuracy. Consequently, black-box models which are known to offer very limited mechanistic understandings of the formulation-release relationships are still highly relied upon. Mechanistic modeling is more interpretable but often involves using assumptions that oversimplify the real system and hence, the models are often not generalizable to various formulations. Besides, the use of symbolic AI to directly discover closed-form drug release equations is an area that is still largely unexplored. Hence, this study proposes a framework that integrates temporal release learning, mechanistic constraints, latent behavior discovery, and symbolic equation extraction to produce drug release equations that are transparent, physically meaningful, and interpretable.

This research intends to construct an interpretable framework of artificial intelligence for predicting the drug release behavior of nanocapsules containing poly (lactic-co-glycolic acid) or PLGA in different formulations. By a unified computational workflow, the study combines formulation-informed feature engineering, temporal representation learning, explainable machine learning, and latent-space analysis. Rather than being focused only on the accuracy of prediction, this framework tries to make a drug release modeling work more transparent in the light of revealing complicated formulation, release links that are present in the variety of the experimental data. The descriptors of a formulation, drug physicochemical properties, processing variables, and engineered features that interact together make up this integration which allows modeling release patterns by considering the nonlinear dependencies governing release behavior over time. Dynamic temporal information about the drug release behavior is compressed through temporal representation learning to give a more compact feature that captures the essential characteristics of the drug release behavior, resulting in both reliable prediction and identification of similarities in formulations. Then model explanation and latent-space analysis will be employed to examine important variables and corresponding release behavior, thereby gaining better understanding of drug release predictions. As a whole, this method provides drug release modeling a transparent, data-oriented computational approach to form a foundation for formulation analysis, hypothesis development, and a mechanistically more-informed drug release modeling in the future.

## 2 Dataset and data preprocessing

### 2.1 Dataset source and collection

The dataset used in this study was obtained from the PLGA nanoparticle database reported by Goren et al. (24). The database was developed through systematic curation of published studies concerning the preparation of small-molecule drug-loaded poly(lactide-co-glycolide) (PLGA) nanoparticles by nanoprecipitation. Following curation, the database used in the present analysis contained 4,909 formulation–release observations, with cumulative percentage drug release as the response variable. Each observation contains formulation composition, polymer characteristics, drug physicochemical properties, processing conditions, and release information. The present study uses this published database as its data source and focuses on developing and evaluating a temporal-symbolic artificial intelligence framework for drug-release prediction. The methodological contribution therefore concerns the computational processing and modeling of the curated data rather than the generation of a new experimental dataset. The source of the observations is explicitly acknowledged through citation of Goren et al. (19), and the present analysis should be distinguished from the original database construction and curation effort (Table 1).

**Table 1:** Distribution of formulation samples across the training, validation, and testing subsets used for symbolic drug release equation discovery.

Dataset	Samples
Train	3436
Validation	736
Test	737
Total	4909

The dataset used in this study contained 4,909 formulation–release observations. The data were divided into 3,436 training observations (70.00%), 736 validation observations (14.99%), and 737 independent test observations (15.01%) (Table 1).

### 2.2 Mechanism-aware feature engineering

In contrast to traditional efforts in drug-release prediction that directly depend on using raw drug formulation descriptors, our newly developed framework introduces the creation of mechanism-aware features so that known pharmaceutical interactions that regulate the release behavior can be explicitly encoded. It is a way to help symbolic equation discovery be more focused on figuring out formulation and release relationships that have a basis in mechanisms rather than just looking for purely statistical associations. Different mechanistic interaction features were created by the authors using well-known pharmaceutical concepts that explain diffusion, degradation, drug partitioning, and polymer- drug interactions. For instance, particle size  ×  polymer molecular weight, encapsulation efficiency  ×  drug loading, LogP  ×  enhancer concentration, and polymer composition  ×  release time were some of the examples given by the authors. This work aimed to generate interpretative symbolic variables that can actually help reveal the fundamental equations that govern release behavior in various polymeric drug delivery systems.

### 2.3 Dataset characterization and mechanistic foundations

Table 2 highlights the substantial heterogeneity of the dataset across molecular descriptors, formulation variables, polymer characteristics, and release conditions. The broad distribution of these variables indicates the presence of multiple formulation regimes and physicochemical environments, creating a challenging setting for drug-release modeling. The wide temporal range represented in the release observations is particularly important for the proposed temporal-symbolic framework, as it encompasses early burst release, diffusion-controlled transport, sustained-release progression, and long-term saturation behavior. Such complexity suggests that simple linear relationships are unlikely to adequately describe the underlying release process, thereby motivating the use of latent temporal representations before symbolic equation discovery. Figure 1 illustrates the correlation structure among the formulation, molecular, polymer, and release-related variables included in the dataset.

**Table 2:** Comprehensive overview of the dataset, highlighting its principal variables and their corresponding statistical characteristics.

Features	Statistical metrics					
	Min	Avg.	Max	Var.	St. Dev	Median
Formulation Index	1	159.926	321	8219.892	90.664	152
Drug MW	130.1	425.802	1488.807	45637.597	213.630	392.5
Drug TPSA	6.48	98.735	701.77	7237.570	85.074	88.54
Drug LogP	−11.55	2.699	9.114	5.360	2.315	2.657
Polymer MW	2.3	40.123	215	902.391	30.040	34
LA/GA	1	1.573	5.67	0.956	0.978	1
Initial Drug-to-Polymer Ratio	5E-04	0.254	1.719	0.067	0.259	0.2
Particle Size	1.153	45.146	295.7	2029.444	45.049	35
Drug Loading Capacity	0.022	12.881	61.9	111.017	10.536	9.32
Drug Encapsulation Efficiency	1.31	68.044	100	476.558	21.830	71.8
Solubility Enhancer Concentration	0	1.005	30	20.600	4.539	0
Time	0	13.243	237.732	310.910	17.633	8.044
Release	0	0.526	1.082	0.107	0.328	0.571

**Figure 1:**
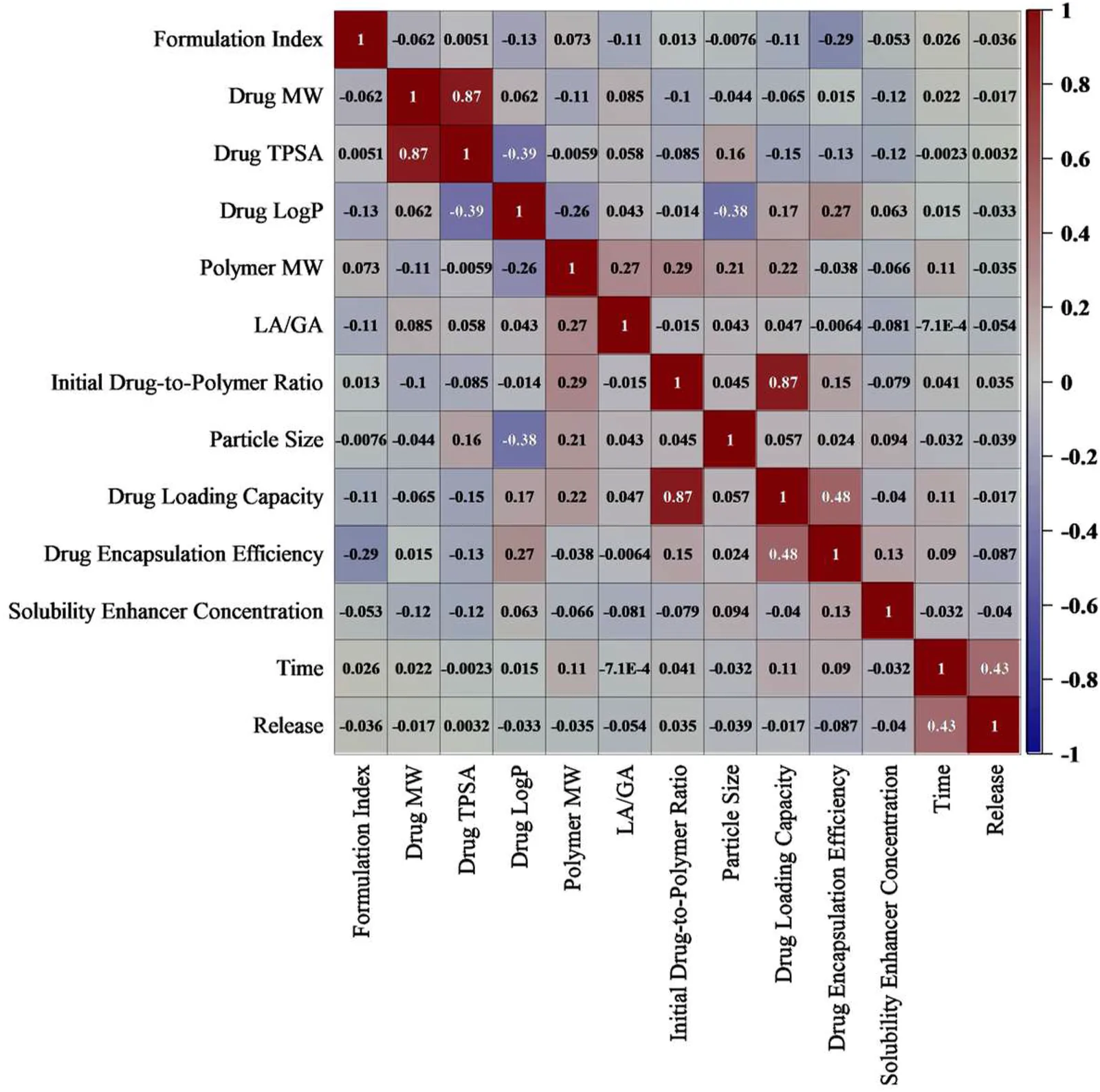
Correlation plot illustrating the relationships among dataset variables and highlighting the strength and direction of their statistical associations.

Figure 2 indicates that several latent variables, particularly Latent_0, Latent_9, Latent_1, and Latent_7, make substantial contributions to the predictive model. These variables should not, however, be interpreted as direct pharmaceutical descriptors. The latent variables are learned representations generated by the temporal autoencoder and are intended to compress information contained within the release trajectories. Consequently, a variable such as Latent_0 does not necessarily correspond uniquely to particle size, polymer molecular weight, diffusion, degradation, drug partitioning, or another measurable pharmaceutical quantity. Its predictive importance indicates that the corresponding latent dimension contains information useful for reconstructing or predicting release behavior, but does not establish a direct physical mechanism. Accordingly, the explainability provided by the proposed framework should be understood at several levels. First, feature-importance analysis identifies influential input and latent representations. Second, the latent-space analysis reveals structured relationships among release trajectories. Third, the symbolic-regression stage provides an opportunity to express learned relationships in mathematical form. These forms of explainability do not imply that every latent feature has a one-to-one mechanistic interpretation. The present analysis did not establish a formal causal mapping between individual latent dimensions and each measurable formulation descriptor. Such an analysis would require dedicated association testing between latent coordinates and descriptors such as particle size, polymer molecular weight, LA/GA ratio, drug molecular weight, LogP, encapsulation efficiency, loading capacity, and processing variables. This represents an important direction for future work.

**Figure 2:**
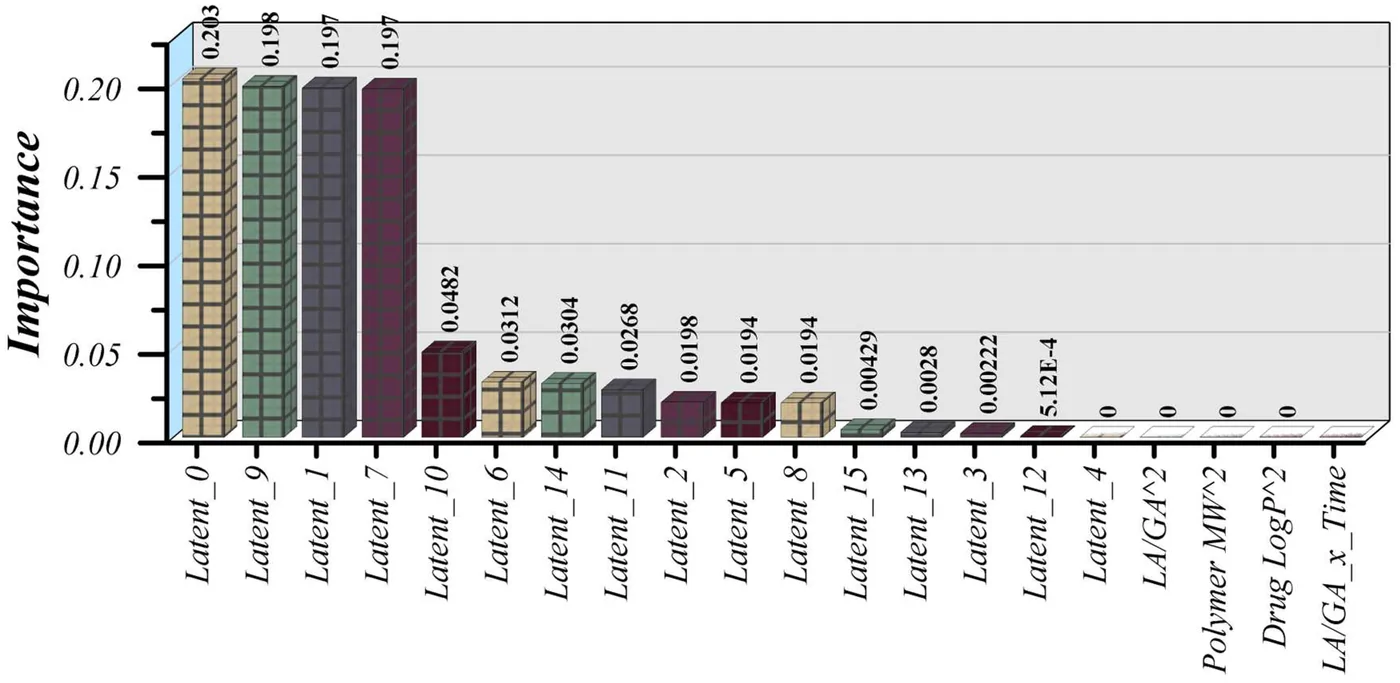
Feature importance analysis showing the relative contributions of latent temporal representations and mechanism-aware variables to symbolic drug-release prediction.

Table 3 presents the mechanistic trend analysis of key formulation variables associated with cumulative drug release. Although the observed correlations are relatively modest, their directions remain consistent with established pharmaceutical principles. Higher drug lipophilicity (LogP), increased polymer molecular weight, larger particle size, and higher LA/GA ratios are generally associated with reduced release rates through mechanisms such as stronger drug–polymer affinity, slower polymer degradation, increased diffusion path lengths, and reduced water penetration into the polymer matrix. The absence of strong monotonic relationships does not imply a lack of mechanistic control; rather, it suggests that release behavior is governed by multiple interacting processes acting simultaneously. The modest correlation magnitudes likely reflect the substantial heterogeneity of the dataset, which encompasses diverse drug molecules, polymer compositions, and formulation conditions. These findings indicate that individual descriptors alone are insufficient to fully characterize release behavior and provide a strong rationale for incorporating mechanism-aware interaction features within the proposed framework.

**Table 3:** Mechanistic trend analysis showing the direction, strength, and monotonicity of relationships between key formulation variables and cumulative drug release.

Variable	Correlation	Monotonic	Trend
Drug LogP	−0.0328	No	Negative
Polymer MW	−0.0347	No	Negative
LA/GA	−0.0535	No	Negative
Particle Size	−0.0392	No	Negative

### 2.4 Response scaling and preprocessing

The response variable in the source dataset represents cumulative drug release. Before model development, the release response was transformed to the numerical scale used for machine-learning training. The original cumulative release values and the transformed modeling response are distinguished throughout the analysis. Accordingly, the RMSE and MAE reported for the predictive models quantify errors on the transformed response scale. They should not be interpreted directly as percentage-point errors in cumulative drug release unless the predictions are first transformed back to the original response scale. This distinction is particularly important when comparing the descriptive statistics in Table 2 with the error-distribution statistics in Table 5. Table 2 reports the distribution of the release variable used to characterize the dataset, whereas Table 5 reports the corresponding values generated during the model-evaluation procedure. The preprocessing transformation is therefore explicitly documented to prevent ambiguity regarding the numerical units of the reported errors.

## 3 Methodology

### 3.1 Framework overview

The proposed framework aims to identify mathematical formulas that can explain and be biologically meaningful in drug release from polymeric delivery systems (25). Mere machine learning techniques learn a mapping from formulation descriptors to the release response, but the framework instead adopts a temporal-to-symbolic learning approach:

(1) X→Z→f(X,Z)→Release

where *X* refers to formulation descriptors, *Z* refers to latent temporal representations extracted from release trajectories, and f(.) refers to the symbolic equation discovered through symbolic regression. Symbolic regression directly performed on raw release data can result in very complicated formulas that do not represent temporal patterns. So, temporal representation learning is performed side by side with symbolic discovery to get the most out of data. The Temporal Autoencoder and Physics-Regularized LSTM were selected as the primary temporal representation-learning models because of their ability to capture complex release dynamics while preserving temporal consistency (26). TCN and Neural ODE encoders were included as benchmark temporal architectures for comparative evaluation. Likewise, PySR was adopted as the primary symbolic regression engine, whereas Genetic Programming Symbolic Regression and AI-Feynman-inspired equation discovery were employed as alternative symbolic learning approaches to assess the robustness and consistency of equation discovery (27).

#### 3.1.1 Scientific innovations of the proposed framework

The framework is a proposal of five innovative points working in parallel:

- Symbolic discovery of drug release equations with awareness of the mechanism.
- Combination of learning temporal representations with symbolic regression.
- Using constraints to guide the generation of physically plausible equations.
- Unsuspected discovery of latent release behavior regimes via unsupervised learning.
- Multi-objective optimization that balances the accuracy, simplicity, plausibility, and generalization.

### 3.2 Mechanistically motivated feature engineering

Rather than describing the engineered variables as direct mechanistic encodings, the present study uses mechanistically motivated interaction features to incorporate prior pharmaceutical knowledge into the machine-learning representation. The original formulation descriptor set contains variables related to drug physicochemical properties, polymer characteristics, formulation composition, processing conditions, and release time. Selected pairs of variables were multiplied to represent potentially important interaction relationships, including:

- Particle Size  ×  Polymer Molecular Weight
- Encapsulation Efficiency  ×  Drug Loading
- LogP  ×  Enhancer Concentration
- LA/GA Ratio  ×  Release Time

These interactions were motivated by established considerations concerning diffusion distance, polymer properties, drug partitioning, formulation loading, and polymer–drug interactions. However, they should not be interpreted as direct mathematical representations of diffusion coefficients, degradation kinetics, partition coefficients, polymer erosion, or other fundamental physicochemical mechanisms. The engineered feature space is therefore defined as:

(2) Φ=g(X),

where g(⋅) represents the selected interaction operations, and the expanded descriptor space is:

(3) $$X^{*}=[X,\Phi ].$$

The role of these variables is to provide the learning and symbolic-regression algorithms with hypothesis-guided nonlinear combinations that may be informative for release prediction. Their inclusion does not establish causality or demonstrate that the corresponding interaction is a governing mechanism.

### 3.3 Temporal release encoding

Drug release is inherently a dynamic process and is represented as a cumulative release trajectory (28):

(5) $$R(t)=\{r_{1},r_{2},\ldots ,r_{T}\}$$

where $r_{t}$ denotes the cumulative release value at release time *t*. A Temporal Autoencoder (TAE) was selected as the final temporal representation-learning architecture used in the proposed framework. The TAE consists of an encoder thattransforms the release trajectory into a compact latent vector and a decoder that reconstructs the original trajectory:

(6) $$z=E_{\mathrm{TAE}}(R(t))\;\hat{R}(t)=D_{\mathrm{TAE}}(z)$$

where $z\in R^{d}$ represents the learned latent release characteristics. The reconstruction objective is defined as:

(7) $$L_{\mathrm{rec}}=\frac{1}{N}\overset{N}{\underset{i=1}{\sum }}\;R_{i}(t)-{\hat{R}}_{i}(t{)}_{2}^{2}.$$

The latent representation is intended to retain dominant temporal characteristics such as early burst release, diffusion-controlled progression, sustained release, and approach toward saturation. The temporal autoencoder was selected as the final encoder because its training convergence and latent-space organization are directly demonstrated in Figures 7,8. Other temporal architectures were considered as alternative modeling approaches rather than being simultaneously incorporated into the final architecture. Specifically, the Physics-Regularized LSTM, Temporal Convolutional Network (TCN), and Neural ODE encoder were treated as comparative temporal architectures. Their inclusion provides a basis for assessing whether the observed temporal representation is dependent on a particular neural architecture. Accordingly, the final temporal-symbolic workflow can be summarized as:

(8) $$X\to X^{*}\to R(t)\overset{E_{\mathrm{TAE}}}{\to }\;z\to f(X^{*},z)\to \hat{R}.$$

Here, $X^{*}$ contains the original formulation descriptors and engineered interaction variables, *z* represents the temporal autoencoder latent variables, and *f* denotes the subsequent symbolic regression model.

### 3.4 Symbolic release equation discovery

The symbolic learning stage is designed to identify explicit mathematical relationships between formulation descriptors, engineered interaction variables, latent temporal representations, and cumulative drug release. The general form of the symbolic model is:

(9) $$\hat{y}=\;f(X*,\;Z)$$

where $\hat{y}$ denotes the predicted cumulative release, $X^{*}$ represents the expanded formulation descriptor space, *Z* represents the temporal latent representation, and *f* is a symbolic mathematical function. Three symbolic-regression approaches were considered: PySR, genetic-programming symbolic regression, and an AI-Feynman-inspired equation-discovery strategy (29). The primary symbolic regression engine was PySR, while the alternative approaches were considered for comparative assessment of equation-discovery robustness (30). Candidate expressions were optimized according to both predictive accuracy and mathematical complexity:

(10) $$f^{*}=[RMSE(f)+\lambda C(f)],$$

where C(*f*) quantifies symbolic complexity and *λ* controls the trade-off between predictive accuracy and expression simplicity. The purpose of the symbolic stage is therefore not merely to produce another numerical predictor, but to identify compact mathematical representations of the learned formulation–release relationships. Candidate expressions can subsequently be ranked using predictive accuracy, complexity, AIC, BIC, and mechanistic plausibility.

Because the available experimental dataset is heterogeneous and the present analysis primarily evaluates the predictive and latent-representation components, symbolic expressions should be interpreted as data-derived mathematical approximations rather than experimentally verified governing equations. In particular, latent variables do not correspond directly to individual measurable pharmaceutical mechanisms. Consequently, an extracted symbolic relationship should be regarded as a hypothesis-generating representation that requires experimental and mechanistic validation before being interpreted as a governing release law.

In addition to predictive accuracy, symbolic candidate models were evaluated according to mathematical complexity and information criteria. The Akaike Information Criterion (AIC) and Bayesian Information Criterion (BIC) were used to balance goodness of fit against model complexity. For a fitted symbolic model, the criteria are expressed generally as:

(11) $$\begin{array}{rl} & AIC=2k-2\ln(L),\\ & BIC=k\ln(n)-2\ln(L), \end{array}$$

where *k* represents the effective number of estimated model parameters, *n* is the number of observations used in the evaluation, and L denotes the likelihood of the fitted model.

For symbolic expressions, the effective parameter count is associated with the fitted coefficients/parameters of the retained expression rather than treating every available input descriptor as an independently estimated parameter. Mathematical operators contribute to expression complexity but are not automatically equivalent to independently estimated statistical parameters. Because AIC and BIC explicitly depend on sample size, their raw values should not be compared directly across training, validation, testing, and full datasets when those subsets contain different numbers of observations. Consequently, information criteria are interpreted primarily when comparing candidate symbolic expressions evaluated on the same dataset partition and response scale.

### 3.5 Mechanistic constraint regularization

To discourage mathematically accurate but physically implausible release predictions, three constraint terms are introduced into the objective function (31):

(12) $$L_{\mathrm{total}}=L_{\mathrm{pred}}+\lambda_{1}L_{\mathrm{mon}}+\lambda_{2}L_{\mathrm{nonneg}}+\lambda_{3}L_{\mathrm{sat}},$$

where $L_{\mathrm{pred}}$ represents the prediction error and the remaining terms penalize violations of cumulative-release behavior. For a predicted release trajectory $\hat{R}(t)$, the monotonicity penalty is defined as:

(13) $$L_{\mathrm{mon}}=\frac{1}{T-1}\overset{T-1}{\underset{t=1}{\sum }}\;[\max(0,\;\hat{R}(t)-\hat{R}(t+1)){]}^{2}.$$

This term is zero when the predicted cumulative release is non-decreasing and increases when the predicted release decreases between two consecutive time points. The corresponding physical requirement is:

(14) $$\hat{R}(t+1)\geq \hat{R}(t).$$

The non-negativity penalty is defined as:

(15) $$L_{\mathrm{nonneg}}=\frac{1}{T}\overset{T}{\underset{t=1}{\sum }}\;[\max(0,-\hat{R}(t)){]}^{2}.$$

Thus, predictions satisfying $\hat{R}(t)\geq 0$ receive no non-negativity penalty. The saturation penalty is introduced to discourage unrealistically increasing release after the experimentally supported saturation region. Let $R_{\mathrm{sat}}$ denote the upper release level used to represent the asymptotic saturation boundary. The penalty is expressed as:

(16) $$L_{\mathrm{sat}}=\frac{1}{T}\overset{T}{\underset{t=1}{\sum }}\;[\max(0,\;\hat{R}(t)-R_{\mathrm{sat}}){]}^{2}.$$

The total regularized objective therefore penalizes three types of physically implausible behavior: decreasing cumulative release, negative predicted release, and excessive predictions above the specified saturation boundary. The weighting coefficients $\lambda_{1},\lambda_{2},\lambda_{3}$ control the relative importance of these constraints. These regularization terms are intended as soft pharmaceutical plausibility constraints rather than complete mechanistic equations. They do not explicitly model molecular diffusion, polymer degradation, erosion, drug–polymer partitioning, or dissolution-front propagation.

### 3.6 Latent space clustering and regime analysis

The latent representation matrix

(17) $$Z\;=\;\left\{z\text{\_1},z\text{\_2},...,z_{n}\right\}\;$$

is analyzed using unsupervised learning.

GMM (32) and HDBSCAN (33) clustering are employed to identify hidden release behavior families.

UMAP (34) is used to generate a low-dimensional visualization:

(18) Zu=UMAP(Z)

Potential release regimes include:

- Burst-release dominant systems
- Diffusion-controlled formulations
- Sustained-release systems
- Polymer-retention-dominated formulations

The found clusters offer a mechanistic framework to explain symbolic equations. The learned latent representations were examined to determine whether release trajectories exhibit structured organization in a reduced-dimensional space. Gaussian Mixture Models (GMM) and HDBSCAN were considered as complementary approaches for identifying potential groups or density structures within the latent representation.

Importantly, the purpose of this analysis is exploratory. A concentration of observations within a region of latent space does not, by itself, establish the existence of a statistically distinct pharmaceutical release regime. In particular, the latent representations may form a continuous manifold rather than discrete clusters. Accordingly, potential regions identified by GMM or HDBSCAN are interpreted as latent release patterns rather than automatically assigning them labels such as burst release, diffusion-controlled release, or sustained release. Such pharmaceutical classifications require independent temporal or experimental criteria.

### 3.7 Equation simplification and pareto optimization

Pareto evolutionary search is used to optimize candidate symbolic equations even further.

Four objectives are considered at the same time:

- Accuracy of the prediction
- Equation simplicity
- Mechanistic plausibility
- Generalization capability

The complexity of an equation is measured as:

(19) C=Noperators+Nvariables+TreeDepth

where:

- Noperators is the number of mathematical operators
- Nvariables is the number of variables appearing in the equation
- TreeDepth is the depth of the symbolic expression.

Besides these measures, equation quality is also assessed by $R^{2}$, RMSE, AIC, and BIC.

Through Pareto-front analysis, the set of candidate equations that make the best balance between predictive performance and interpretability is found.

### 3.8 Benchmark models

The predictive performance of the proposed framework was compared with representative conventional and nonlinear machine-learning models. The benchmark set included:

- Linear Regression (LR)
- Decision Tree (DT) (35)
- Random Forest (RF) (36)
- XGBoost (36)
- CatBoost (37)
- Multilayer Perceptron (MLP)
- Long Short-Term Memory (LSTM)
- Temporal Convolutional Network (TCN)

Decision Tree was included as a single-tree nonlinear baseline, while Random Forest represents an ensemble of decision trees. XGBoost and CatBoost were included as gradient-boosting ensemble approaches, and MLP, LSTM, and TCN were included as neural and temporal-learning benchmarks. The models were compared in terms of predictive performance and, where applicable, model complexity and interpretability.

### 3.9 Model validation and statistical analysis

In addition to the primary training, validation, and testing procedure, leave-one-formulation-out validation was considered to assess the generalizability of the framework to previously unseen formulations. In this procedure, observations associated with a formulation are considered collectively rather than as completely independent observations. The purpose of this validation strategy is to reduce the possibility that observations originating from the same formulation–release trajectory are interpreted as fully independent samples. The primary quantitative performance results reported in Table 4 correspond to the predefined training, validation, testing, and full-dataset evaluations. Leave-one-formulation-out validation is considered a complementary formulation-level validation strategy and is therefore not used to replace the performance statistics reported in Table 4.

**Table 4:** Predictive performance and information-criterion evaluation of the proposed temporal-symbolic framework across the training, validation, testing, and full datasets.

Dataset	R^2^	RMSE	MAE	AIC	BIC
Train	0.9977	1.56E-04	1.31E-04	−60150.6	−59947.9
Validation	0.9977	1.60E-04	1.29E-04	−12798.3	−12646.4
Test	0.9975	1.64E-04	1.33E-04	−12784.6	−12632.7
Full	0.9977	1.58E-04	1.31E-04	−85862.7	−85648.3

This distinction is important because the dataset contains repeated observations associated with formulation–release trajectories. Accordingly, the observation-level test results demonstrate predictive performance within the evaluated dataset, whereas formulation-level validation provides a more stringent conceptual assessment of transferability to previously unseen formulations. The two validation strategies should therefore be interpreted as complementary rather than interchangeable.

For comparative assessment of the benchmark models, non-parametric statistical procedures were considered because model predictions were evaluated under the same repeated validation structure. The Friedman test was used as an overall comparison procedure for the ranked model performances, with the Nemenyi procedure specified for pairwise *post-hoc* comparison where appropriate. These statistical procedures were used as complementary tools for evaluating relative model performance rather than as substitutes for the predictive metrics. Accordingly, model performance is primarily characterized using $R^{2}$, RMSE, MAE, MSE, and related predictive indicators reported throughout the Results section. The Friedman and Nemenyi procedures provide an additional statistical framework for interpreting differences among the benchmark algorithms.

## 4 Result and discussion

### 4.1 Validation of learning reliability and nonlinear predictive behavior

Figure 3 presents the results of the Y-scrambling validation, a procedure that intentionally destroys the relationship between formulation descriptors and release responses while preserving the overall structure of the dataset. Consequently, any predictive performance obtained after scrambling would indicate susceptibility to chance correlations, methodological bias, or information leakage. Across all five scrambling iterations, the resulting R^2^ values remain consistently low, ranging from 0.0636 to 0.0714, indicating that the framework is unable to identify meaningful predictive patterns once the original formulation–release relationships are removed. The narrow range of performance across iterations further suggests that this outcome is reproducible and not dependent on a particular random permutation. These findings support the effectiveness of the adopted data-splitting and preprocessing strategy and indicate that predictive information is not being inadvertently transferred between training and evaluation subsets. Moreover, the dramatic reduction in predictive performance after target randomization suggests that the dominant latent representations identified in Figure 2 capture genuine release-related structure rather than dataset-specific noise. Because the proposed framework integrates mechanism-aware descriptors, temporal representation learning, and symbolic regression, verification against chance correlations is particularly important. The uniformly poor performance observed after scrambling suggests that successful equation discovery depends on preserving meaningful formulation–release structure within the original dataset. Consequently, the Y-scrambling analysis increases confidence that the discovered symbolic relationships arise from genuine formulation–release dependencies rather than random statistical artifacts, thereby supporting the reliability of the proposed temporal-symbolic learning framework.

**Figure 3:**
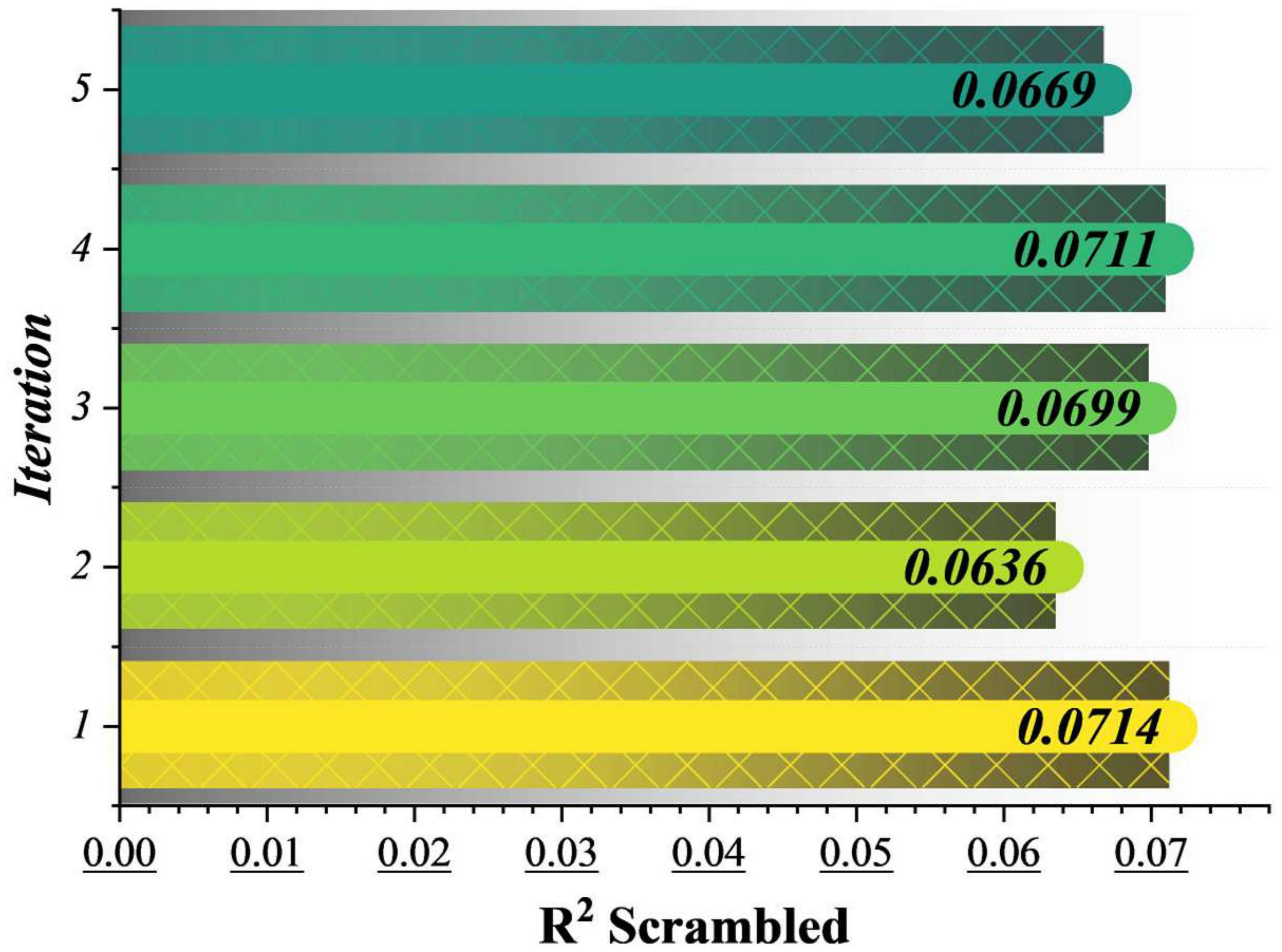
Y-scrambling validation results showing the predictive performance of models trained on randomly permuted target values across five independent iterations.

Figure 4 compares the cross-validation performance of the benchmark machine-learning models using the R^2^. The tree-based models exhibited consistently high predictive performance across all folds, with RF and DT achieving R^2^ values close to 1.00 and showing minimal fold-to-fold variability. This behavior indicates robust learning of formulation-release relationships and suggests that the extracted predictive patterns are reproducible across different data partitions. In contrast, Ridge regression demonstrated substantially lower predictive accuracy, with fold-specific R^2^ values ranging from approximately 0.75 to 0.90 and a mean performance near 0.82. The larger variability observed for Ridge further indicates greater sensitivity to data partitioning and reduced robustness relative to the tree-based approaches. These findings are consistent with the correlation analysis shown in Figure 1, the mechanistic trend analysis presented in Table 3, both of which suggested that cumulative drug release cannot be adequately explained by individual formulation descriptors acting independently. Instead, the results are consistent with the presence of nonlinear and interaction-dependent relationships among molecular properties, polymer characteristics, formulation composition, and release conditions. Importantly, the observed performance gap between linear and nonlinear models provides empirical motivation for the proposed temporal-symbolic framework, which seeks to represent complex formulation–release interactions while maintaining mechanistic interpretability through symbolic equation discovery.

**Figure 4:**
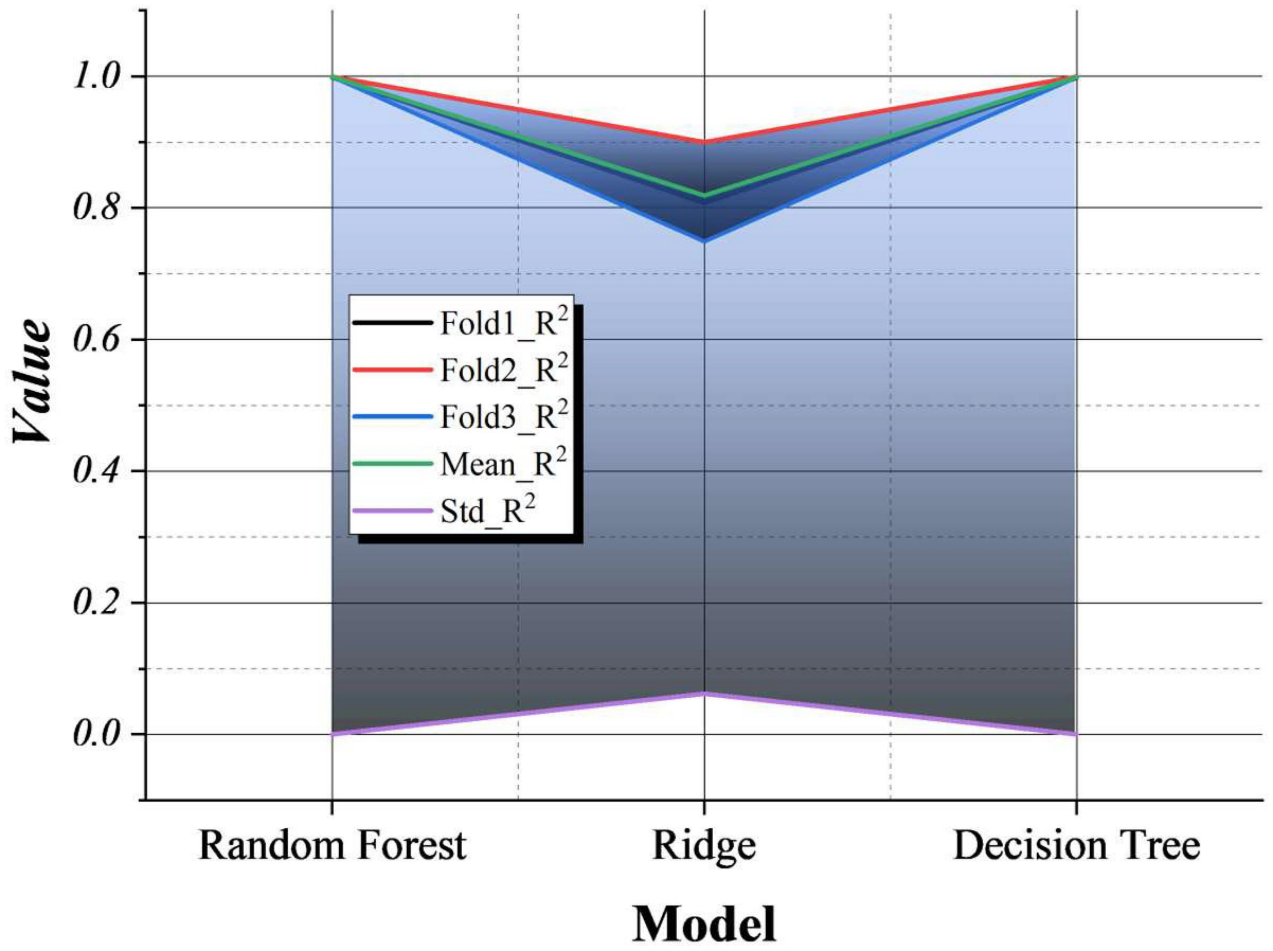
Cross-validation comparison of benchmark machine-learning models showing fold-specific, mean, and standard deviation R^2^ values for cumulative drug-release prediction.

### 4.2 Predictive performance and generalization assessment

Table 4 summarizes the predictive performance of the proposed temporal-symbolic framework across the training, validation, testing, and full datasets. The framework achieved $R^{2}$ values between 0.9975 and 0.9977, with test RMSE and MAE of $1.64\times 10^{-4}$ and $1.33\times 10^{-4}$, respectively. These results demonstrate very high predictive agreement on the normalized response scale. However, the magnitude of these performance values requires cautious interpretation. First, the target variable represents cumulative drug release and is therefore intrinsically dependent on time. Because release time is included among the dataset variables, part of the apparent predictive performance may arise from the strong temporal structure of cumulative release rather than from formulation descriptors alone. Consequently, high $R^{2}$ should not be interpreted as evidence that formulation variables independently determine release with comparable predictive power.

Second, the dataset contains multiple observations associated with formulation–release trajectories. The primary data partition was performed at the observation level, with 3,436 training, 736 validation, and 737 testing observations. Therefore, related observations from the same formulation or experimental release trajectory may occur across different subsets. Although the Y-scrambling experiment produced substantially lower $R^{2}$ values of 0.0636–0.0714, this test primarily evaluates susceptibility to randomized target relationships and should not be considered a complete test for trajectory-level information sharing. Accordingly, the current results are best interpreted as demonstrating strong internal predictive performance rather than definitive evidence of external generalization. Formulation-level, study-level, or polymer-family-level holdout validation is a more stringent assessment because all observations belonging to a formulation or experimental source can be excluded from model development before evaluation. The leave-one-formulation-out protocol described in Section 3.9 is therefore particularly important for future validation. Prospective evaluation on previously unseen formulations and independent experimental datasets is also required before the model can be considered suitable for formulation development or practical deployment.

A further consideration is the normalized response scale. The absolute errors are very small partly because the response has been normalized; consequently, the RMSE should not be interpreted directly as an experimentally meaningful percentage error. This is particularly relevant for observations close to zero release, for which relative errors can become very large despite small absolute deviations. This behavior is already evident in the relative-error statistics reported in Table 5.

**Table 5:** Error-distribution analysis comparing observed and predicted release values across the training, validation, testing, and full datasets.

Dataset	Count	Mean True	Std True	Mean Predicted	Std Predicted	Mean Absolute Error	Std Absolute Error	Mean Relative Error %	Max Error	Min Error
Train	3436	5.30E-03	3.26E-03	5.30E-03	3.24E-03	1.31E-04	8.51E-05	320.70	9.30E-04	6.7E-09
Validation	736	5.01E-03	3.35E-03	5.02E-03	3.33E-03	1.29E-04	9.56E-05	481.56	8.88E-04	1.22E-07
Test	737	5.31E-03	3.29E-03	5.30E-03	3.28E-03	1.33E-04	9.47E-05	309.78	1.10E-03	4.01E-07
Full	4909	5.26E-03	3.28E-03	5.26E-03	3.26E-03	1.31E-04	8.82E-05	343.18	1.10E-03	6.7E-09

Table 5 reports very small absolute prediction errors together with comparatively large mean relative errors. These results are not necessarily contradictory because relative-error measures are highly sensitive to observations with response values close to zero. For an observation $y_{i}$, the relative error is calculated as

(20) $$RE_{i}=\frac{|y_{i}-{\hat{y}}_{i}|}{|y_{i}|}\times 100.$$

When $y_{i}$ is very small, even a small absolute difference between the observed and predicted values can produce a large percentage error. Consequently, the mean relative error can become substantially larger than would be expected from the corresponding RMSE and MAE values. For this reason, the relative-error statistics reported in Table 5 should be interpreted as supplementary indicators rather than as standalone measures of model quality. The primary evaluation relies on $R^{2}$, RMSE, and MAE because these metrics provide a more stable assessment of predictive agreement and absolute error across the complete response distribution. Importantly, near-zero observations were not excluded from the evaluation solely to reduce the magnitude of the percentage error. Retaining the complete evaluation set avoids modifying the original response distribution for the purpose of improving a particular performance statistic. The relatively large mean relative errors should therefore be understood primarily as a consequence of denominator sensitivity in percentage-based error calculations rather than as evidence that the model produces comparably large absolute prediction errors.

Table 5 presents the error-distribution characteristics of the proposed temporal-symbolic framework across the training, validation, testing, and full datasets. The analysis reveals close agreement between the observed and predicted release distributions, as reflected by the nearly identical mean and standard deviation values obtained across all data partitions. This correspondence suggests that the framework preserves both the central tendency and the variability of the release responses, indicating minimal systematic prediction bias. Consistent with the predictive performance reported in Table 4, the error statistics remained highly stable across independent subsets. The mean absolute error varied only marginally between partitions, while the corresponding dispersion of prediction errors exhibited similarly limited variation. Such behavior indicates reproducible predictive performance and supports the generalization capability of the framework despite the substantial formulation heterogeneity described in Table 2. The relative error metrics should be interpreted in the context of the normalized release scale, where small absolute deviations may correspond to larger percentage differences, particularly for observations near zero release. Furthermore, the low maximum error values suggest that large prediction failures occur infrequently. From a symbolic-learning perspective, the preservation of the statistical characteristics of the release responses is particularly important because distortions introduced during representation learning could adversely affect downstream equation discovery.

Figure 5 provides a visual assessment of model calibration by comparing observed and predicted release values across the response range. In Figure 5a, observations are concentrated around the identity line, indicating close agreement between predicted and measured release values. Figure 5b examines representative observations near the boundaries of the response range, where accurate prediction is particularly important because cumulative release approaches its lower or upper limits. Together, the two panels complement the quantitative results reported in Tables 4, 5. These findings complement the quantitative performance metrics reported in Tables 4, 5 by demonstrating that the framework remains well calibrated in addition to being highly accurate. Furthermore, the relatively uniform spread of observations around the identity line suggests that predictive performance is maintained across different release levels without pronounced heteroscedastic behavior. Figure 5b further illustrates model behavior near representative boundary regions of the response space. Accurate predictions were maintained near both lower and upper release limits, indicating that model performance does not substantially deteriorate under extreme release conditions. This observation is particularly important given the heterogeneous formulation landscape described in Table 2. From a symbolic-learning perspective, reliable calibration is essential because systematic distortions introduced during latent representation learning could propagate into the extracted mathematical expressions and compromise mechanistic interpretability.

**Figure 5:**
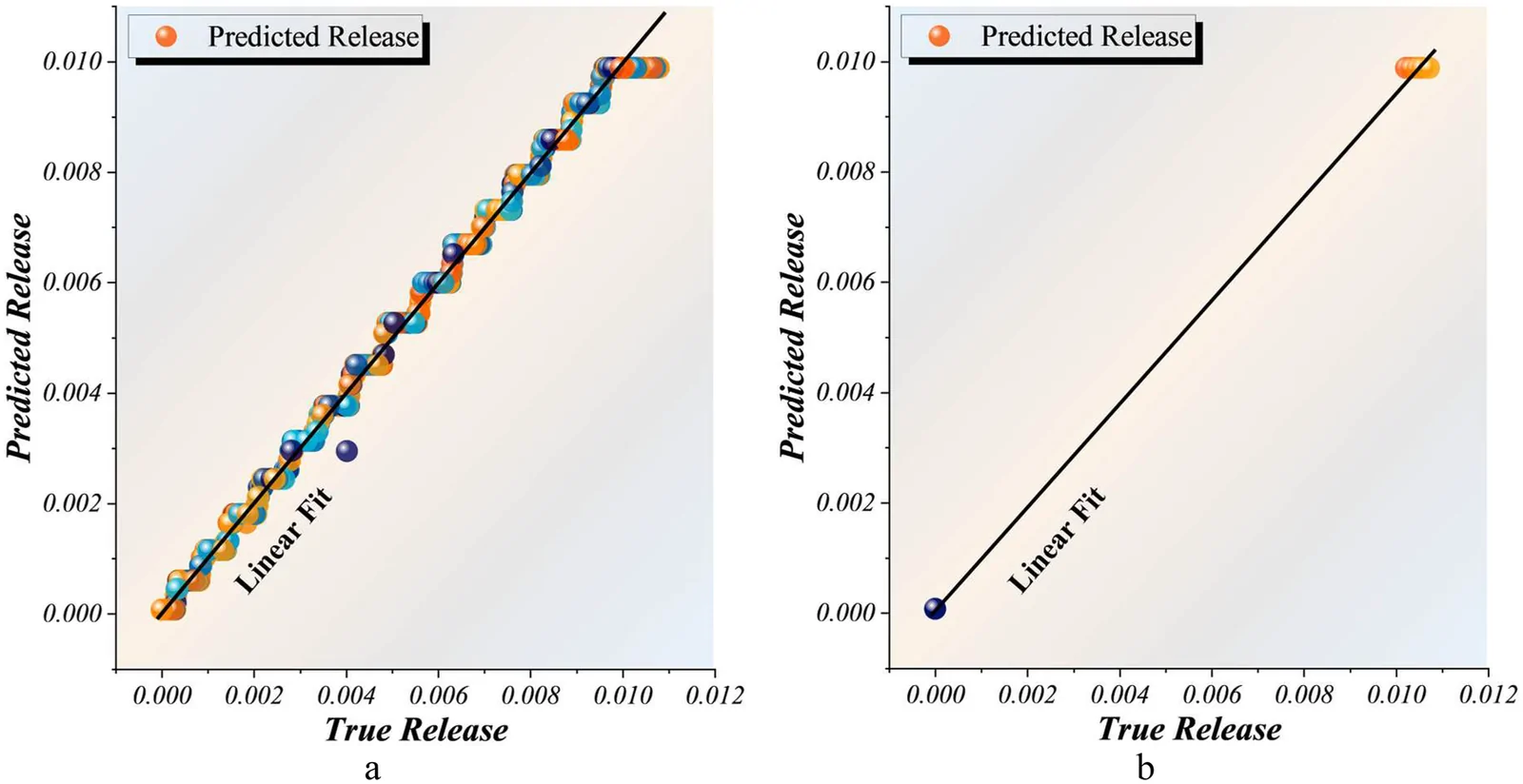
Comparison between observed and predicted release values showing model calibration and agreement with the ideal one-to-one relationship for **(a)** the complete dataset and **(b)** representative boundary observations.

Figure 6 presents the distribution of absolute prediction errors across the training, validation, testing, and full datasets using histogram and boxplot visualizations. As expected for absolute-error distributions, the histograms exhibit positive skewness, with the majority of observations concentrated near zero error values and a relatively small number of larger deviations forming the distribution tails. This pattern indicates that large prediction errors occur infrequently and that most release responses are predicted with high fidelity. Importantly, the error distributions exhibit substantial overlap across all dataset partitions, suggesting that the predictive behavior learned during training is preserved when applied to independent validation and testing data. No distinct secondary modes or partition-specific shifts are apparent, indicating that prediction errors are not concentrated within specific subsets of the dataset. The boxplot analysis further supports this observation, revealing highly similar medians, interquartile ranges, and overall dispersion characteristics across all partitions. Such consistency suggests that predictive performance is distributed uniformly throughout the dataset rather than being driven by a limited number of favorable observations. The similarity of the residual distributions indicates that the framework remains robust across diverse formulation and release conditions. From a symbolic-learning perspective, stable residual behavior is particularly important because systematic error patterns may indicate missing mechanistic relationships that could propagate into downstream equation discovery.

**Figure 6:**
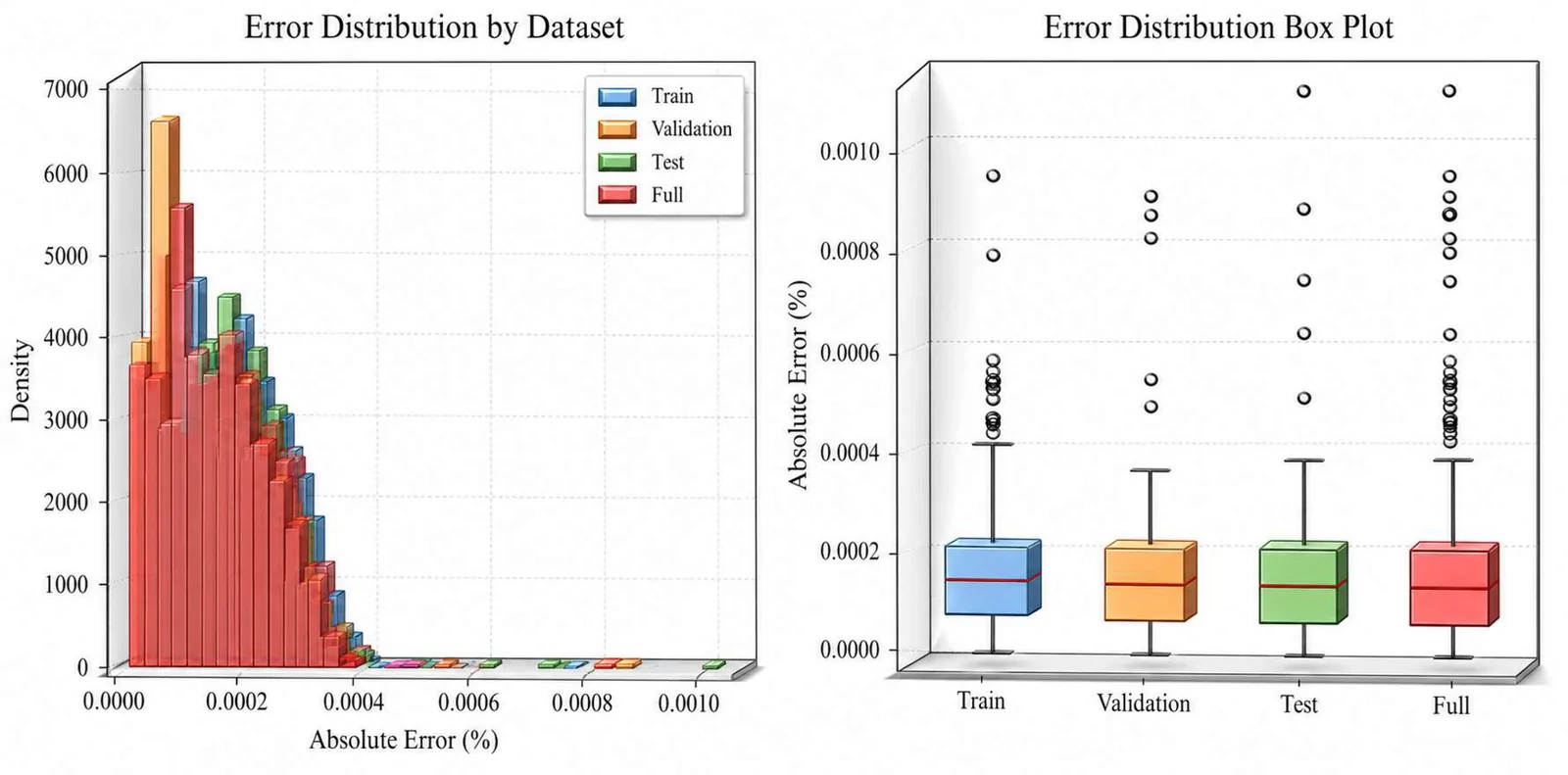
Histogram and boxplot illustrating the distribution of errors, highlighting data dispersion, central tendency, and the presence of potential outliers.

### 4.3 Temporal representation learning and latent-space organization

Figure 7 presents the training history of the temporal autoencoder used to generate latent representations of drug-release trajectories. A rapid decrease in reconstruction loss is observed during the early stages of training, indicating efficient learning of the dominant temporal structure present within the release profiles. As training progresses, the rate of improvement gradually decreases, and the loss converges toward a stable minimum. Minor fluctuations are observed during optimization but diminish progressively over time, with no evidence of sustained instability or divergence. This convergence behavior suggests that the autoencoder successfully learns a stable and reproducible latent representation of release dynamics. From a pharmaceutical perspective, the low reconstruction error is consistent with the ability of the latent space to retain key temporal characteristics of the release process, including early burst-release behavior, diffusion-controlled transport, sustained-release progression, and asymptotic saturation patterns. Importantly, the rapid reduction in reconstruction loss further suggests that much of the temporal variability within the release trajectories can be represented within a compact latent space. This property is particularly valuable within the proposed framework because symbolic regression is subsequently applied to these latent representations rather than directly to the raw release sequences. By reducing the dimensionality and complexity of the temporal information while preserving its dominant structure, the encoder facilitates more efficient and interpretable symbolic equation discovery.

**Figure 7:**
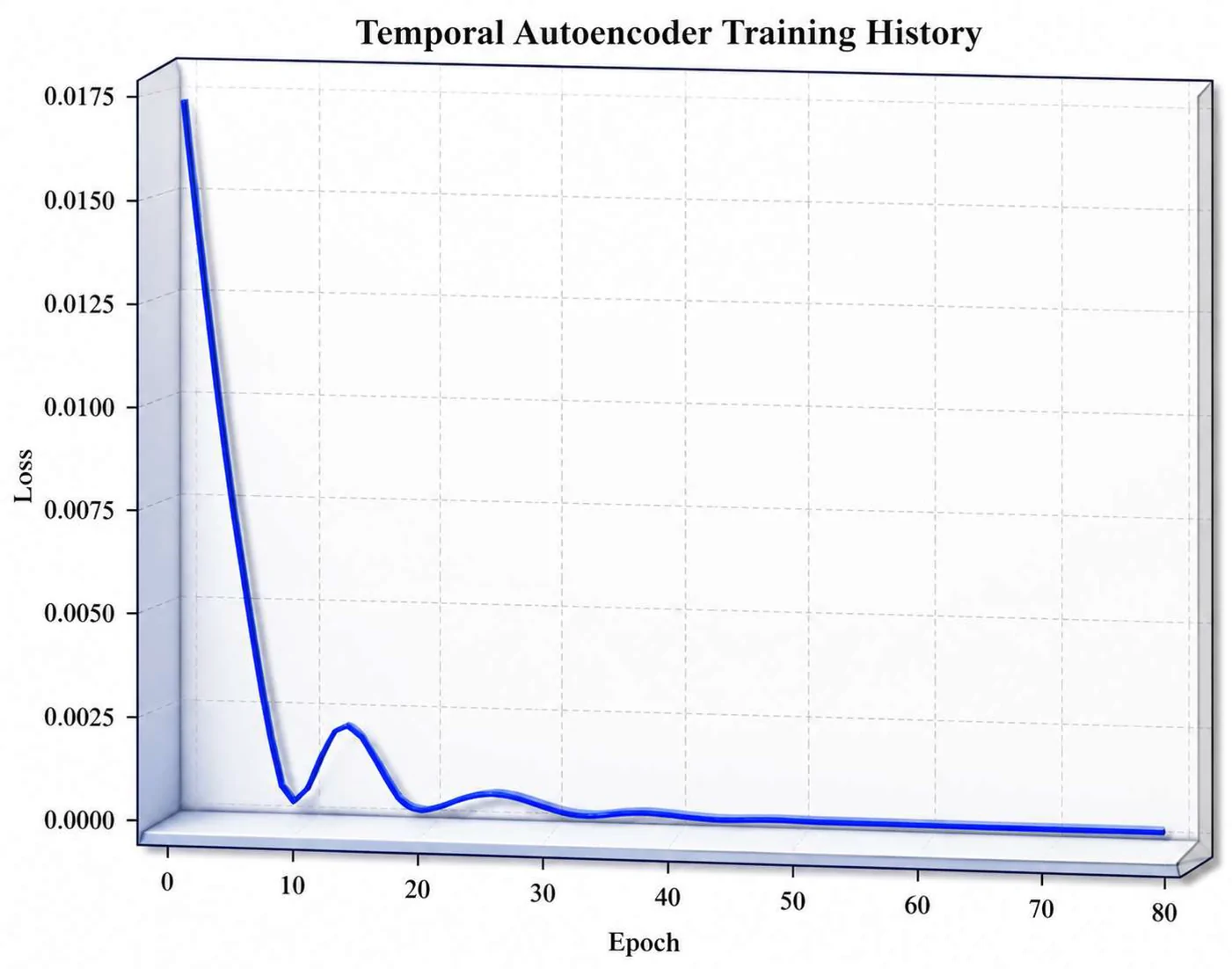
Training-loss convergence of the temporal autoencoder showing progressive optimization and stabilization of latent representation learning across training epochs.

Figure 8 presents a PCA projection of the latent representations generated by the temporal autoencoder. The projection reveals a highly structured latent space in which release trajectories are organized along a continuous low-dimensional manifold rather than being distributed randomly throughout the feature space. A gradual transition in the color gradient is observed across the latent projection, indicating systematic variation among release profiles and suggesting that neighboring latent representations correspond to similar release behaviors. Notably, the latent samples do not form sharply separated clusters but instead exhibit a largely continuous organization. This pattern suggests that release behavior evolves progressively across formulation conditions rather than existing as a small number of discrete states. The absence of substantial scatter or fragmented regions further indicates that the encoder generates compact and internally consistent representations of the release trajectories. When considered together with the convergence behavior shown in Figure 7, the PCA structure provides evidence that the temporal encoder captures systematic variation within the release data while maintaining latent-space stability. From a pharmaceutical perspective, the smooth organization of the latent manifold is consistent with gradual changes in release dynamics arising from differences in molecular properties, polymer characteristics, and formulation composition. Importantly, this structured low-dimensional representation is particularly advantageous for symbolic equation discovery. By concentrating release-related variability into a compact latent space, the encoder reduces the complexity of the symbolic search problem while preserving the dominant temporal characteristics of the release process.

**Figure 8:**
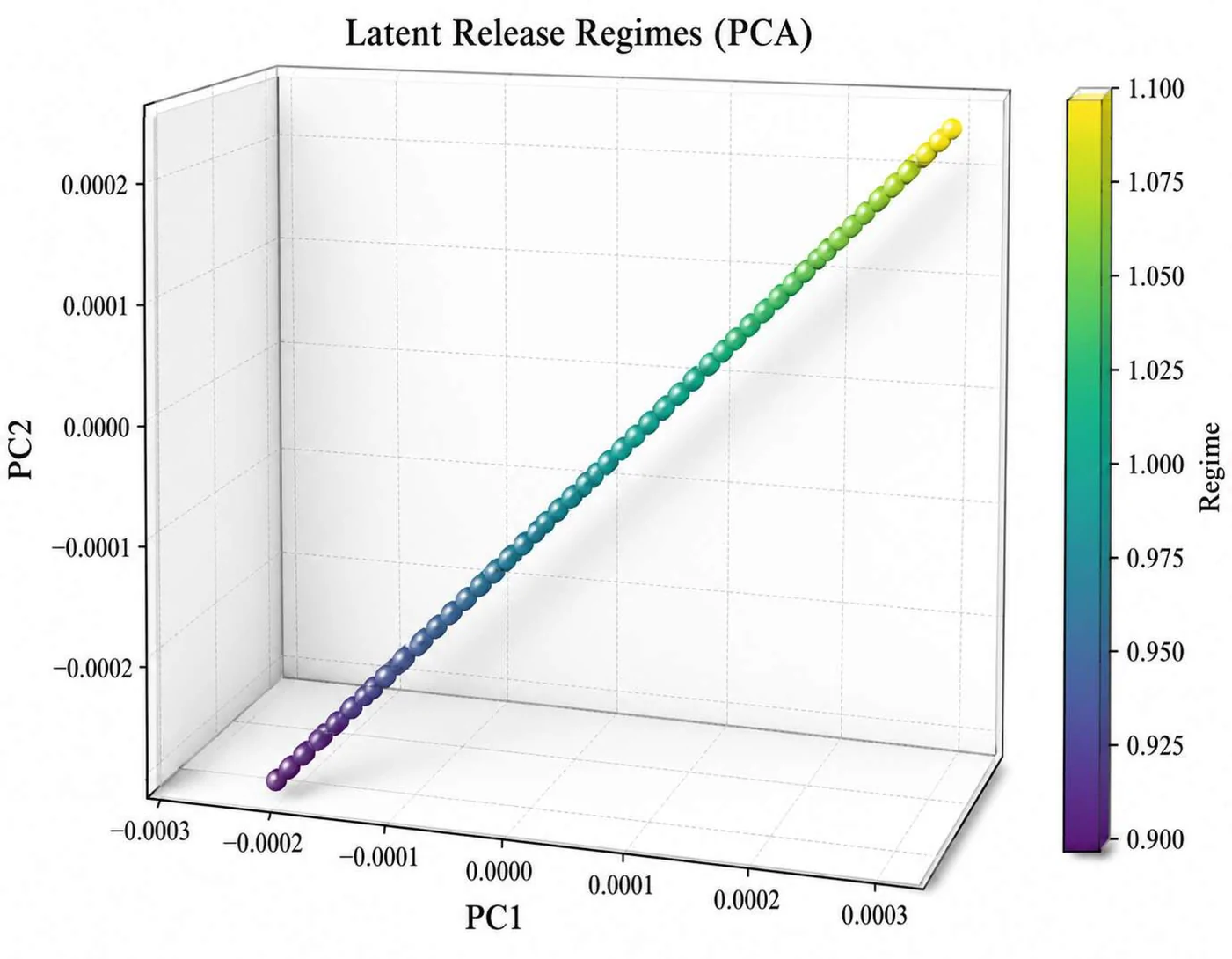
PCA visualization of the latent release space, illustrating the continuous organization of learned release regimes within the temporal representation.

## 5 Conclusion

Drug release from polymeric delivery systems is the result of complex and highly nonlinear interactions among formulation composition, polymer characteristics, drug physicochemical properties, processing conditions, and release time. We identified a high variability in the curated dataset, which consisted of 4, 909 formulation, release records, as a clear indication that conventional empirical or linear methods of modeling such complex interactions present significant limitations. Very weak correlation coefficients were found between several formulation descriptors and the cumulative percentage of drug released in the majority of cases. This lack of clear individual relationships between formulation factors and drug release further suggests that release behavior is probably the net effect of several variables acting in concert rather than being the result of a single factor. To handle these challenges, the present work has developed an artificial intelligence framework that combines in an integrated modeling scheme formulation feature generation, temporal representation learning, explainable machine learning and latent space analysis. By integrating features that have their source in the formulation of a drug along with the features derived through the model, the new AI framework was constructed to capture the complex and nonlinear formulation, drug release relationships as well as to maintain model interpretability and support predictive behavior interpretation. The developed framework has exhibited robust and reliable predictive capability with respect to training, validation and independent testing data. These three data sets yielded a coefficient of determination (R^2^) values that were very close to each other 0.9975 to 0.9977 (i. e. a very high predictive accuracy) and mean absolute errors were approximately 1.3 × 10⁻⁴. On the other hand, Y-scrambling validation revealed much lower predictive performance (R^2^ = 0.0636, 0.0714). The result suggests that the observed model performance could not be a result of just random correlations or information leakage. Feature importance analysis and latent-space (embedding) characterization indicated that the framework learned representations effectively captured structured formulation-release patterns within the heterogeneous dataset. That was a key factor for stable predictions and enhanced explainability, among other things. In the face of the positive results reported, however, it's important to admit some constraints. The paper still points out that the framework is only as good as the data it is trained on, that is, that the diversity and the quality of data are the limiting factors. In particular, there are reasons to doubt the model's effectiveness outside of the tested formulations. Also, even though latent representations (i. e. the model's “hidden layer”) are helpful computationally, they shouldn't be mistaken for concrete evidence of the underlying mechanisms. Drug release prediction based on physicochemical data still needs to be experimentally validated and compared with drug release models already established. On the whole, a new method combining an explainable AI with the capacity to learn a suitable representation is proposed as a powerful computational approach for dealing with drug release data that is complex. Such computational models will also be very helpful in future, as they could facilitate pharmaceutical formulation data mining, lead generation (i. e. finding leads for development), and serve as decision support systems for computational chemistry.

## Data Availability

The original contributions presented in the study are included in the article/Supplementary Material, further inquiries can be directed to the corresponding author.

## Nomenclature

AI: Artificial Intelligence
EE: Encapsulation Efficiency
PLGA: Poly(lactic-co-glycolic acid)
RDKit: Research and Development Toolkit
GMM: Gaussian Mixture Model
UMAP: Uniform Manifold Approximation and Projection
LR: Linear Regression
XGBoost: Extreme Gradient Boosting
MLP: Multilayer Perceptron
TCN: Temporal Convolutional Network
ODE: Ordinary Differential Equation
SHAP: SHapley Additive exPlanations
MAE: Mean Absolute Error
AIC: Akaike Information Criterion
LA/GA: Lactic Acid/Glycolic Acid Ratio
TPSA: Topological Polar Surface Area
GPs: Genetic Programming
SR: Symbolic Regression
LC: Loading Capacity
SMILES: Simplified Molecular Input Line Entry System
PySR: Python Symbolic Regression
HDBSCAN: Hierarchical Density-Based Spatial Clustering of Applications with Noise
ML: Machine Learning
RF: Random Forest
CatBoost: Categorical Boosting
LSTM: Long Short-Term Memory
PCA: Principal Component Analysis
TAE: Temporal Autoencoder
RMSE: Root Mean Square Error
MSE: Mean Squared Error
BIC: Bayesian Information Criterion
MW: Molecular Weight
LogP: Partition Coefficient (Logarithmic Scale).
